# What do we know about grant peer review in the health sciences?

**DOI:** 10.12688/f1000research.11917.2

**Published:** 2018-03-27

**Authors:** Susan Guthrie, Ioana Ghiga, Steven Wooding

**Affiliations:** 1 RAND Europe, Westbrook Centre, Milton Road, Cambridge, UK; 2 Centre for Science and Policy, University of Cambridge, Cambridge, UK

**Keywords:** peer review, grant awarding, funding allocation, grant reviewing

## Abstract

**Background**: Peer review decisions award an estimated >95% of academic medical research funding, so it is crucial to understand how well they work and if they could be improved.

**Methods**: This paper summarises evidence from 105 papers identified through a literature search on the effectiveness and burden of peer review for grant funding.

**Results**: There is a remarkable paucity of evidence about the efficiency of peer review for funding allocation, given its centrality to the modern system of science. From the available evidence, we can identify some conclusions around the effectiveness and burden of peer review.

The strongest evidence around effectiveness indicates a bias against innovative research. There is also fairly clear evidence that peer review is, at best, a weak predictor of future research performance, and that ratings vary considerably between reviewers. There is some evidence of age bias and cronyism.

Good evidence shows that the burden of peer review is high and that around 75% of it falls on applicants. By contrast, many of the efforts to reduce burden are focused on funders and reviewers/panel members.

**Conclusions**: We suggest funders should acknowledge, assess and analyse the uncertainty around peer review, even using reviewers’ uncertainty as an input to funding decisions. Funders could consider a lottery element in some parts of their funding allocation process, to reduce both burden and bias, and allow better evaluation of decision processes. Alternatively, the distribution of scores from different reviewers could be better utilised as a possible way to identify novel, innovative research. Above all, there is a need for open, transparent experimentation and evaluation of different ways to fund research. This also requires more openness across the wider scientific community to support such investigations, acknowledging the lack of evidence about the primacy of the current system and the impossibility of achieving perfection.

## Introduction

Health research has contributed enormously to society, but it is also expensive. This has led to increasing demands to understand and improve how research is supported. Most effort has focused on evaluating impacts of research, on society and the economy. Funders are attempting to gather evidence of impact using online survey platforms such as Researchfish in the UK, and national assessment frameworks including Excellence for Research in Australia (ERA).

Much less work has focused on understanding how research is selected for support. Peer review is used to allocate the vast majority of competitive research funding internationally ( Ismail *et al.* (2009) estimated that >95% of UK medical research funding was allocated by peer review). Therefore it is crucial to understand whether peer review is effective and efficient - whether it can fairly, reliably allocate research funding without bias. In this study, we carried out a rapid evidence assessment which asked whether the peer review process lives up to these aspirations.

The research was commissioned by the Canadian Institutes of Health Research (CIHR) to support an ongoing review of CIHR’s peer review system, particularly the Peer Review Expert Panel which was convened to review the design and adjudication processes of CIHR’s investigator-initiated research programmes.

## Methods

### Search strategy

We identified relevant literature through five routes, using 2009 as our cut-off date because this was the date of our previous review ( Ismail *et al.*, 2009):

1. Google Scholar search using the search terms below, for publications from 2009 onwards. We reviewed the top 500 search results for each query.

Search terms:

– ‘Grant peer review’
– ‘Grant review’ AND ‘panel’
– (‘Peer review’ AND ‘funding application’) OR (‘peer review’ AND proposal) OR (‘peer review’ AND funding) OR (‘peer review’ AND award) or (‘peer review’ AND ‘reviewer bias’)

2. Grey literature: we searched the websites of major funding bodies and other academic bodies (e.g. learned societies) that we expected to have published relevant research ( Table 1).

**Table 1.** Academic bodies considered in the review of literature.

Organisation	Country
National Institutes of Health (NIH)	USA
Canadian Institutes of Health Research	Canada
Health Research Board of Ireland	Ireland
Science Foundation of Ireland	Ireland
Netherlands Organisation of Health Research and Development (ZonMw)	Netherlands
Research Council of Norway	Norway
National Institute for Health Research (NIHR)	UK
Wellcome Trust	UK
National Health And Medical Research Council	Australia
Health Research Council of New Zealand	New Zealand
Medical Research Council	UK
Deutsche Forschungsgemeinschaft (DFG)	Germany
Lundbeck Foundation, Copenhagen	Denmark
Swedish Medical Research Council	Sweden
Swedish Society for Medicine	Sweden
European Commission	European Union

3. Searching the Cochrane publication list for systematic reviews on grant peer review. This did not identify any relevant reviews conducted since 2009.

4. An initial set of publications already known to the authors and sponsors of the work.

5. Snowballing: from the reference lists of publications identified following screening.

Some elements of our strategy were focused on evidence from the health sciences (particularly grey literature), but our wider searches, including Google Scholar, were not restricted by field of research.

### Screening strategy

Publications were initially screened on title, and abstract (where available). Studies needed to include empirical consideration of the effectiveness and/or burden of grant review processes. Studies were excluded on the basis of being:

– Purely descriptive, describing a specific peer review process.
– Focused on wider concerns around the funding process, with no (or only tangential) reference to the peer review process in particular.
– Focused on manuscript peer review rather than peer review for funding purposes.
– From 2008 or earlier.
– Reviews, with no additional synthesis or analysis, summarising work from before 2008, or studies already identified and included individually.

If studies were relevant full text was retrieved and an Excel spreadsheet was used to capture key information on the study and its conclusions.

We identified 105 studies for inclusion. Table 2 summarises the range of studies identified. At the suggestion of our reviewers we added five additional references ( Bollen *et al.*, 2017; Bromham *et al.*, 2016; Doran *et al.*, 2014; Höylä *et al.*, 2016; Kulage *et al.*, 2015), we also added the term (‘fellowship’ AND ‘peer review’) to our final Google Scholar search and reviewed the top 100 results adding two further references ( Ginther *et al.*, 2011; Kurokawa *et al.*, 2015).

**Table 2.** Breakdown of articles included in the review.

Number of studies included		105
Type of document	Peer-reviewed publication	70
	Other articles from journals, not peer reviewed (e.g. comment pieces)	22
	Grey literature	8
	Working paper	1
	Book chapter	4
Format of document	Commentary	21
	Review	15
	Empirical study	69
Type of data used (empirical studies)	Quantitative	53
	Qualitative	12
	Mixed methods	4
Subject focus	Biomedical	13
	Wider health	45
	Wider research	47
Quality of studies (GRADE)	0 (Lower quality, e.g. commentary with no or limited evidence base)	14
	1 (Triple-downgraded randomized trials, downgraded observational studies, or case series/case reports)	35
	2 (Double-downgraded randomized trials or observational studies)	38
	3 (Downgraded randomized trials or upgraded observational studies)	17
	4 (Randomized Trials or double- upgraded observational studies)	1

**Table 3.** Summary of evidence from the literature regarding the effectiveness and burden of peer review.

Evaluation question	General critique	Particular criticism(s)	Is the criticism valid?	Strength of the evidence base
Is peer review an effective system for awarding grants?	Peer review does not fund the best science	It is anti-innovation	Yes	Suggestive
		It does not reward interdisciplinary work	Unclear	Suggestive
		It does not reward translational/ applied research	Unclear	Suggestive
		It is only a weak predictor of future performance	Yes	Agreement
	Peer review is unreliable	Ratings vary considerably betwee reviewers	Yes	Agreement
		It struggles to achieve an acceptable level of consistency	Unclear	Conflicting
	Peer review is unfair	It is gender-biased	Unclear	Conflicting
		It is age-biased	Unclear	Conflicting
		It is biased by cognitive particularism	Unclear	Conflicting
		It is open to cronyism	Yes	Agreement
	Peer review is not accountable	Review anonymity reduces transparency	N/A	N/A
	Peer review is not timely	It slows down the grant award process detrimentally	Unclear	Suggestive
	Peer review does not have the confidence of key stakeholders	It is not the preferred method of resource allocation	No	Agreement
What is the burden of peer review on the research system?	Peer review is an overly burdensome way of distributing research funding	Burden of peer review is increasing	Yes	Agreement
		Burden of the peer review system is high and falls primarily on the applicants	Yes	Agreement

### Assessment of evidence quality

Quality of evidence was rated on a scale of 1–4 based on GRADE ( Guyatt *et al.*, 2008) ^1^. We aggregated the overall strength of the evidence for each area of criticism based on the scale in Box 1.

Box 1. **Scale for quality of evidence**

1. Assumptions: Intuitive assumptions and widely shared beliefs prevail
2. Suggestive: There is insufficient evidence to draw a clear conclusion (but the evidence is at least suggestive)
3. Conflicting: There are conflicting results from well-conducted studies
4. Agreement: A number of well-conducted studies agree
5. Compelling: Systematic reviews are compelling.

When synthesising our findings, we also drew on our previous review of the topic ( Ismail *et al.*, 2009).

## Results

We summarise our findings in Table 3 with each discussed in detail below.

### Is peer review an effective system for awarding grants?

***The meaning of ‘best’ science is not fixed.*** What constitutes the ‘best’ science will vary, however it may include research that is innovative, interdisciplinary and applied. This section considers biases against any particular type of research and whether peer review is a good predictor of future success.

***Peer review is probably anti-innovation.*** Braben (2004) has suggested that supporting highly innovative research is important because it drives technological change and economic growth – an idea increasingly embraced by research funders. NIH has expressed concern at falling numbers of innovative or risky applications, suggesting ‘competitive pressures have pushed researchers to submit more conservative applications’ ( Kaplan, 2005; Scarpa, 2006). Low success rates may have exacerbated the situation, inducing ‘conservative, short-term thinking in applicants, reviewers, and funders’ ( Alberts *et al.*, 2014). On the other hand, a system is necessary to distinguish between innovative research and that grounded in ‘reckless speculation’ ( Hackett & Chubin, 2003). Although ‘innovative research’ and ‘high-risk research’ are often conflated, they are not necessarily synonymous, here we include both aspects of innovation.

Innovative proposals may have less preceding work supporting them, and hence receive less praise from reviewers ( RIN, 2010; Spier, 2002). This lack of preceding work requires less risk-averse mind-set from the reviewer ( Spier, 2002). Innovative proposals from young researchers may suffer a ‘double disadvantage’: lacking previous work, both because of their novelty and the researcher’s shorter track record.

The challenge of supporting innovation is not new, in 1977, Thomas Kuhn wrote of an ‘essential tension’ between originality and tradition. These tensions were also included in a 2006 UK Treasury report which noted ‘the UK is still susceptible to a charge of risk aversion, as classic peer review criteria emphasise tests of scholarship over potential impact’ ( Treasury, 2006, p. 16). Empirical evidence of this problem come from recent work identifying lower scoring of novel proposals, even controlling for factors such as proposal quality, further this deficit could not be explained by the novel proposals being less feasible ( Boudreau *et al.*, 2012; Boudreau *et al.*, 2016).

Risk aversion may also affect the preparation of applications: Fang & Casadevall (2009) suggested that falling success rates lead to conservatism because of the perceived increased risk associated with innovative proposals.

Approaches to these problems include using reviewers with different cognitive biases for different schemes – specifically targeting specialists in translational or high-risk, innovative research ( Langfeldt, 2001). This approach has been used (though not evaluated) in NIH’s high-risk, high-reward Pioneer awards ( Gewin, 2012).

Making ‘innovation’ an assessment criteria is another approach ( Lindner *et al.*, 2016; Luukkonen, 2012). Views on this are mixed, some suggesting panels lack the expertise to assess innovation ( Costello, 2010), whilst others see the approach as effective ( Spiegel, 2010). Analysis of NIH application scores suggests that those for innovation are closely correlated with overall scores ( Lindner *et al.*, 2016).

Other analysis ( Giraudeau *et al.*, 2011; Linton, 2016), suggests that disagreement among scoring could be used to identify innovative research – high disagreement being taken as an indicator of work with high potential but also high risk. Similarly, Lee (2015) suggests combating conservatism by increasing the weight given to criteria – such as innovation – which are typically underweighted by reviewers.

An approach that sidesteps the issue is to select researchers purely on their merit, regardless of the research they plan to conduct. Researchers then have freedom to pursue new and novel ideas and work flexibly, as opportunities arise (e.g. by the MacArthur Fellows programme ^4^).

Finally, Holliday & Robotin (2010) suggest that a Delphi process (a structured deliberative process) could be used to assess the merits of research ‘in situations where the available scientific evidence is limited and if review panels have widely divergent opinions’. The process was also found to be efficient and flexible from a time perspective.

***It is not clear if peer review treats interdisciplinary research fairly.*** Critics argue interdisciplinary research is disadvantaged because (1) interdisciplinary proposal reviews may have to combine multiple distinct understandings of ‘quality’ – undermining the strength of the review ( Feller, 2006), and (2) it is more difficult to identify ‘peers’ to review such work. This latter challenge is exacerbated by the standard structure of peer review processes in which only a few reviewers examine each proposal in detail, or at the initial stages, reducing the breadth of reviewing expertise further ( Gluckman, 2012). Bromham *et al.* (2016) analyse 18,476 submissions to the Australian Research Council’s Discovery Programme and show that increased interdisciplinarity leads to lower success rates.

A study on the US National Science Foundation (NSF) revealed that, in interdisciplinary studies at least, peer review favours ‘research that is performed by academics, in the sciences, and that falls completely within the reviewers’ own domain of expertise’ ( Porter & Rossini, 1985, p. 37). With interdisciplinary teams it can be hard to isolate the contribution of each researcher, which can reduce the investigators chance of getting further funding by ‘weakening’ their track record ( Cooksey, 2006a)

There has been limited further work in this area since 2009. Increasing the size of the review panel and broadening the range of expertise and disciplines present has been suggested as a way to address these problems. However, this increases burden and can only work if the role of the initial in-depth reviewer(s) is diminished ( Gluckman, 2012).

***It is not clear if peer review fairly assesses applied research.*** The Cooksey Report on health research funding in the UK noted that peer review ‘can in some instances inhibit programmes in translational and applied health research’ ( Cooksey, 2006b). The report suggested that one reason for this inhibition was because peer review prevented the iterative development of research projects where funder and researcher worked together. Cooksey also suggested that because applied researchers publish in specialist (i.e. lower-impact) journals, they received less credit for publications than basic researchers. Including research users and considering the likely impact of research as part of the funding process may address these concerns. In our 2009 review, we noted the Canadian Health Services Research Foundation pioneering work through the use of ‘merit review panels’ to evaluate proposals, combining members from both academic and wider user/policy communities. This approach has now spread to other major funders, notably NIHR. Considering impact at the application stage – an approach criticised for disadvantaging innovative research – is likely to be beneficial when reviewing research which is closer to being applied.

The evidence around peer review’s bias against applied research is not strong and has changed little since 2009. It is hard to know what criteria individual reviewers apply, as studies are hampered by methodological problems and funders are reluctant to release scores from peer review panels ( Feller, 2006). While several studies have examined how reviewers assess proposals in the humanities and social sciences ( Guetzkow *et al.*, 2004; Mansilla, 2006), work in the natural sciences is lacking. A study of NIH shows the success rate of clinical research proposals is marginally less than those for laboratory research ( Kotchen *et al.*, 2004). This is in line with a recent CIHR study showing that health services and policy research applications were less successful than biomedical research applications ( Tamblyn *et al.*, 2016).

***Peer review is at best only a weak predictor of future performance.*** Work by Fang & Casadevall suggests peer review can ‘winnow’ out bad research proposals ( Fang & Casadevall, 2012). However, recent studies from several NIH Institutes and the Netherlands have challenged the idea that peer review can effectively select the best research. Studies comparing percentile application rankings with the research’s subsequent bibliometric performance found no association ( Danthi *et al.*, 2014; Danthi *et al.*, 2015; Doyle *et al.*, 2015; Fang *et al.*, 2016; Kaltman *et al.*, 2014; van den Besselaar & Sandström, 2015). Two further such studies found that grant review outcomes only weakly predict bibliometric performance ( Lauer *et al.*, 2015; Reinhart, 2009). Bibliometric analyses are by no means perfect measures of performance – only capturing a proxy of academic performance ( Belter, 2015). Nonetheless, the findings suggest that peer review assessment is, at best, a crude predictor of performance.

Using an alternative metric, Galbraith *et al.* showed that peer reviewers’ opinions were only weakly predictive of the commercial success of early stage technologies in small businesses ( Galbraith *et al.*, 2010).

Fang & Casadevall (2012) comment that, while reviewers can usually identify the top 20–30 per cent of grant applications, going further to identify the top 10 per cent is ‘impossible without a crystal ball or time machine’ (p.898).

### Is peer review reliable?

If peer review is reliable, the judgements of different peer reviewers on the same proposal should be highly correlated. The grounds for the continuing use of peer review would be severely undermined if systematic unreliability were demonstrated. Funders have been criticised for not making sufficient efforts to measure and monitor the reliability of assessments across reviewers ( Fang & Casadevall, 2009). In this section, we consider two concerns surrounding peer review, namely individual reviews and overall consistency of decisionmaking – and how they might be addressed.

***It is clear that ratings vary considerably between reviewers.*** *Single*-rater reliabilities ^5^ are not encouraging, but have been hampered by the methodological difficulties of modelling the complex interactions between reviewers in multi-stage peer review processes. In particular, the work of Jayasinghe *et al.* (2003) demonstrates a single-rater reliability correlation of just 0.21 for the humanities and social sciences, and an even lower correlation of 0.19 for the sciences. Similarly, Fogelholm *et al.* found an inter-rater reliability of around 0.23 for medical research ( Fogelholm *et al.*, 2012). In contrast, two studies have found a higher level of agreement between reviwers. The first study which built in some of the complexities of the peer review process, found a dependent reliability ^6^ rating for individual peer reviewers of 0.80. The second study on the review process for Marie Curie Actions (a major EU funding stream) measured inter-rater reliability based on the average deviation in scores between raters, and found a high level of agreement ( Pina *et al.*, 2015).

Strikingly, the chance of improvements from initial ratings during panel discussion is virtually nil (e.g. from ‘no award’ or ‘possible award’ to ‘award’). This suggests that initial triage of applications may be preferable to re-rating rounds (Bornmann, *et al.*).

Increasing diversity of background and discipline of peer reviewers also reduces rating consistency. Lobb *et al.* (2013) identified a low intra-class correlation coefficient (0.12) when comparing reviewers from a research, practice or policy background. They also noted that the level of agreement among experts from different disciplines was considerably lower than that among adjudicators of the same discipline, meaning that the presence of several practitioners from the same discipline area could have the potential to skew funding outcomes, depending on the wider makeup of the panel. This suggests that peer review processes may not work well for transdisciplinary teams integrating both academic and non-academic experts. Taking a different perspective, Reinhart found that although the global intra-class correlation coefficient was 0.41, there were considerable differences between fields, for example, biology (0.45) versus medicine (0.20) ( Reinhart, 2009).

***There is conflicting evidence on whether peer review can achieve acceptable levels of decisionmaking consistency.*** Existing studies offer mixed judgements on the reliability of grant peer review. Bornmann identified a threshold of 80–90 per cent as the expectation for agreement for this kind of decisionmaking ( Bornmann *et al.*, 2008). Two early studies ( Cole *et al.*, 1981; Hodgson, 1997) we noted in 2009 found reliability rates across funding boards of 75 and 73 per cent respectively for funding decisions which they felt was a satisfactory level of agreement. More recent evidence is mixed. The most recent study comparing the outcome of two independent panels found an agreement rate of 83 per cent ( Clarke *et al.*, 2016), whilst a previous study in 2012 was less favourable, showing agreement levels of 65–69 per cent ( Fogelholm *et al.*, 2012).

Graves *et al.* (2011) examined the variability of panel members’ individual scores and calculated how this translates into the variability of overall proposal ranking, and hence funding decisions. They found that such variability could affect the outcome for 29 per cent of the proposals considered, and that variability differed widely between panels. Abdoul *et al.* have suggested that scoring variability might be partially explained by differences in reviewer behaviour, such as the time taken to do the assessment, assessment methods, and variation in the relative weighting of different criteria by different reviewers ( Abdoul *et al.*, 2012).

Recent studies focusing more on the impact of panel meetings have shown very limited effects on improving consistency and reliability. Fogelholm *et al.* (2012) suggested that mean reviewer scores prior to the panel meeting were similar to the panel consensus score. The authors concluded that using the mean reviewers’ scores was a practical and economical alternative. Similarly, although Pina *et al.* (2015) identified both a subset of panels and subset of proposals with high levels of disagreement, where consensus meetings improved agreement, across the whole population they could not detect an overall improvement in agreement.

In contrast, Martin *et al.* (2010) found meeting discussions had an important effect in more than 13 per cent of applications in their analysis of a sample of standard (R01) NIH research grant applications.

Two funders have experimented with, and evaluated, virtual peer review both by teleconference and through the use of Second Life, a virtual world. NIH estimated that using Second Life telepresence, peer review could cut panel costs by one third ( Bohannon, 2011). Pier *et al.* (2015) compared videoconference and face-to-face panels. They set up one videoconference and three face-to-face panels modelled on NIH review procedures, concluding that scoring was similar between face-to-face and videoconference panels. Both the Bohannon and Pier studies of virtual panels noted that participants valued the social aspects of meeting in person and preferred the face-to-face arrangements.

Gallo *et al.* (2013) examined four years of peer review discussions, two years face-to-face and two years teleconferencing. They found minimal differences in merit score distribution, inter-rater reliability or reviewer demographics. They also noted that panel discussion, of any type, affects the funding decision for around 10 per cent of applications relative to original scores.

***Approaches to improve reliability have been tried.*** The NIH peer review self-study suggested some possible improvements to the peer review process to combat low reliability, focusing principally on better training for reviewers ( NIH, 2008). NIH suggested such training should focus on: (1) emphasising the strengths (rather than weaknesses) of research proposals; (2) focusing on the potential impact of research; (3) reviewing the merit of the proposal and not re-writing it; (4) recognising the problem of implicit bias in study sections; (5) using benchmark applications during panel meetings to provide review guidelines; and (6) pointing out potential bias towards lesser known applicant organisations.

Recent work by Sattler *et al.* (2015) has evaluated the effect this type of brief training programme. The study found inter-rater reliability increased from 0.61 to 0.89, and the amount of time spent reviewing also increased, for both new and experienced reviewers.

If inconsistency stems from discrepancies in review quality (which is by no means clear), it might be feasible to evaluate the quality of reviews, although this approach has its own challenges – for example, what is a ‘good’ review? If a review is not consistent with other review does that intrinsically make it ‘bad’? It could be the outlier picking up on the true potential of an innovative application. However, this approach is used by many funders, as shown in a report by the European Science Foundation (2011) which found in a survey of European research funders that more than half (60 per cent) evaluate the quality of all reviews as standard practice using a range of criteria (e.g. completeness, level of substantiation, appropriateness, comprehensibility, timeliness and usefulness), and may return the review to a reviewer or reject the review. Organisations felt that review quality was higher where these checks were made, but noted little difference quality between cases where all reviews are evaluated versus just a sample. However, no data was available to assess these suggestions, and no empirical analysis had been carried out. Adding such an evaluation process clearly adds to the burden of the process.

***Is peer review fair?*** Having considered the evidence suggesting that consensus on peer review decisions is rare, what factors might underlie the observed discrepancies? To what extent is peer review open to the same allegations of bias that plague science more widely, particularly around gender, race, intellectual school or institutional affiliation? A recent study ( Day, 2015) has shown that low levels of passive bias as well as individual cases of significant active bias among reviewers can have significant impacts on the outcomes of a grant peer review process, earlier work showed the likely presence of racial bias in NIH funding decisions ( Ginther *et al.*, 2011). In this section we consider the potential for bias in peer review across four main areas: gender, age, cronyism and cognitive particularism.

Bias could occur at various places in the peer review process. While bias on the part of the peer reviewers themselves (such as sexism or racism) has received considerable attention in the literature, funding competitions can be biased through eligibility and award selection criteria. Such criteria may be prejudiced against early career researchers or innovative research – although there is no strong evidence that this occurs. In addition wider systemic biases may mean that the number of applications received is lower from particular groups.

Blinding of applications provides a defence against the most obvious abuses by reviewers – rejecting proposals on the grounds of race, gender, institutional affiliation and so forth ( Lee *et al.*, 2012). A study from South Korea by Lee *et al.* (2000) demonstrated a significant bias in sighted proposal evaluation towards those from particular research departments, senior researchers, and those already academically recognised. This is reinforced by a review of studies by the NSF, which found only ‘a weak correlation’ between panel ratings of blinded short version and unblinded full versions of the same applications ( Bhattacharjee, 2012). While some funding bodies now routinely attempt to anonymise proposals *before* passing them on to reviewers, there is some dispute as to whether anonymisation is truly possible. Some authors contend that some degree of identification is always possible from anonymised research proposals ( Bhattacharjee, 2012)

***There is a substantial body of conflicting evidence on whether peer review is gender biased.*** The overall demographics of science - with increasing under-representation of women as seniority levels increase point to particular challenges for women in advancing in science. However, the evidence on gender bias in peer review is inconclusive. Studies suggesting bias include an important study of the grant peer review system of the Swedish Medical Research Council strongly suggested that reviewers were unable to judge scientific merit independently of gender ( Wenneras & Wold, 1997). These findings were supported by a subsequent meta-analysis of 21 studies on this topic, which found that grant applications submitted by men were 7 per cent more likely to be approved than those submitted by women ( Bornmann *et al.*, 2007). ^7^ Furthermore, recent studies have also found evidence of gender bias ( Jang *et al.*, 2016; Kaatz *et al.*, 2014; Kaatz *et al.*, 2015; Tamblyn *et al.*, 2016; van der Lee & Ellemers, 2015; Volker & Steenbeek, 2015). For example, van der Lee & Ellemers (2015) reported a 4 per cent ‘loss’ of women during the grant review process for awards to early career scientists by the Netherlands Organization for Scientific Research (NWO). In a review of research on gender bias by Kaatz *et al.* (2014), women generally have lower rates of publication and lower success rates for high-status research awards than do men.

On the other hand, a review of the gender bias literature by Ceci & Williams (2011) showed that the weight of evidence suggests that peer review is fair across gender, with all smaller-scale studies analysed, along with all but one of the large-scale studies, failing to replicate Wenneras & Wold’s findings. And even for the remaining large-scale study the findings were reversed by a reanalysis. The lack of gender bias has been supported by several subsequent studies, in particular a careful, large-scale primary study and meta-analysis by Marsh *et al.* ( Marsh *et al.*, 2011; Mutz *et al.*, 2012; Reinhart, 2009; Turner *et al.*, 2014; Van Arensbergen *et al.*, 2013).

***There is a small conflicting evidence base on whether peer review is biased by age.*** Although review processes that partly rely on the previous publications or funding successes of the applicant may be biased against early career researchers, Jayasinghe *et al.* (2001); Jayasinghe *et al.* (2003) found that the age of the applicants did not directly impact upon grant success, a findings supported by Reinhart (2009). However, this finding is directly contradicted by a study comparing sighted and blinded reviews of research grant proposals in South Korea ( Lee *et al.*, 2000). A subsequent study, also based in South Korea ( Jang *et al.*, 2016), found that evaluation scores and selection success rates decline with age.

Concerns about age bias are closely tied to concerns about bias against early career researchers, who may be disadvantaged through lacking preliminary results or a substantial portfolio of work. The challenges of providing adequate support for early career researchers is widely recognised ( Bazeley, 2003) and was raised in a 2008 NIH review which identified significant decreases early career success rates which could not be accounted for by variations in application quality ( NIH, 2008). Similar concerns were also noted by Spiegel (2010), who showed that the average age researchers won their first full NIH project grant awards (R01) had been steadily increasing. Since then, the NIH has introduced measures aimed at equalising success rates for new and established investigators for new (not renewal) applications.

***There is evidence that peer review suffers from cronyism.*** Cronyism is a concern for many major funders, who have detailed conflict of interest processes in place to counter the presence or perception of such biases. However, ( Wenneras & Wold, 1997) show that prior affiliation with a reviewer considerably increased a researcher’s chances of funding, Similarly, a large-scale study of applications to the National Science Foundation of Korea found that applications reviewed by previous or current affiliates were more likely to be successful ( Jang *et al.*, 2016). A review of NSF proposals reported by Bhattacharjee (2012) is harder to interpret when full proposals and shorter, anonymised versions of the same proposals were compared there were only weak correlations. Panelists and applicants suggested anonymisation made a difference, but the shorter length of proposals was also seen as important.

Luukkonen (2012) notes that panel debate may fail to counter crude forms of cronyism since panels often cover a wide area of research, and each specific area is only represented by a few experts, so the other members may defer to the experts’ knowledge. Members of funding panels may also benefit directly from their membership. One study noted that panel members submit more applications, and have more grant awards ( van den Besselaar, 2012). The challenge in this area is separating factors such as good researchers who submit more applications being selected to join panels or having a better sense of what makes a good application, from nepotism.

***There is conflicting evidence on whether peer review demonstrates cognitive particularism (favouring your own field or way of thinking).*** The idea that reviewers and panel members will favour proposals in their own fields or that align with their ways of thinking has been termed ‘cognitive particularism’ ( Travis & Collins, 1991). Fang & Casadevall (2009), suggest that ‘reviewer biases favour topics well understood and appreciated by the [funding panel]’ (p.930). Travis & Collins (1991) found that reviewers tend to favour proposals supporting their own school of thought, and argues that this is likely to have a much bigger impact on the direction of science than institutional bias or cronyism identified by other studies ( Langfeldt, 2006; Wenneras & Wold, 1997). Research by Li (2015) suggests the same. Work by Wang & Sandström (2015) suggests that ‘cognitive distance’ may influence reviewer decisions in a more complicated way, with reviewers more likely to favour applications in areas they are either very familiar with, or completely unfamiliar with. Other studies find that reviewers are more critical of applications in areas of their own expertise ( Boudreau *et al.*, 2016; Gallo *et al.*, 2016).

A number of studies suggest that studies in molecular biology are more likely to be successful in comparison to other fields of bioscience. ( Bornmann & Daniel, 2006) found a slight statistical effect and further studies reveal that peer-reviewed grant proposals in molecular biology tend to have a better chance of receiving grant funding than proposals in other bioscience fields ( Kotchen *et al.*, 2004; Taylor, 2001).

There is also dispute about how to resolve this potential problem Alberts *et al.* (2014) suggests that such effects could be countered by broadening ‘the range of scientific problems judged by each group and include[ing] a diversity of fields on each panel’, suggesting that ‘senior scientists with a wide appreciation for different fields can play important roles by counteracting the tendency of specialists to overvalue work in their own field’ (p.5777). However, Li (2015) advises caution, noting that though evaluators may be biased in favour of projects in their own area, they are also likely to be better able to assess the quality of those projects, and the benefits of this expertise may well outweigh any possible biases.

### Is peer review timely?

***There is suggestive evidence that the peer review process slows, and hinders, the progress of research.*** In some cases such as an emerging epidemic the time taken by peer review could reduce the number of people benefiting from the research, such slowing of the research process could also reduce the economic viability of a new product, (e.g. Agres, 2005; Cures, 2005; Daniels, 2004; Roy, 1985). The many stages of grant peer review can take from 9 to 18 months from submission to funding. It is less clear how often this time significantly hinders the progress of science. In the health sciences, research is one of many steps in develop new treatments and practices ( Hanney *et al.*, 2015). Research suggests that the time required for translation of research from initial idea to adopted practice is around 17 years, so peer review may be a relatively small contributor, however any one translation pathway may have multiple stages of peer review ( Morris *et al.*, 2011).

***There is good evidence that peer review has the support of most major scientific stakeholders.*** Though criticism of the peer review process abounds, empirical evidence, though limited, indicates that support for peer review amongst the academic community remain strong ( Bornmann, 2011; Wooding & Grant, 2003). The dominance of peer review across funding systems internationally suggests it has the confidence of institutional stakeholders. A recent review of literature about the NIH peer review processes found a firm belief in the transparency and objectivity of peer review amongst grant reviewers ( Miner, 2011). There is a striking disconnect between the institutional and community support for the peer review system and the empirical evidence of its effectiveness – unfortunately the scope of our review excluded the types of research that might explain this divergence.

In contrast, beyond the classical model of research an emerging body of literature suggests traditional academic peer review may not be appropriate for all types of research. A recent study on indigenous research showed the competitive nature of peer review was counterproductive and that peer review did not have the confidence of relevant stakeholders ( Street *et al.*, 2009). Similar concerns have been expressed about the assessment of community engagement proposals ( Ahmed & Palermo, 2010).

### What is the burden of peer review on the research system?

***The burden of peer review is increasing.*** In a survey of 28 biomedical research funding organisations across 19 countries ( Schroter *et al.*, 2010), declined review requests, late reports and administrative burden were the most frequently mentioned challenges, and all organisations reported an increase in burden in the previous five years (although they reported that the quality of reviews had remained the same). A study by the Royal Society of New Zealand reported a similar increase in the difficulty of recruiting senior reviewers ( Gluckman, 2012).

***The burden of the peer review system is high and falls primarily on the applicants.*** The overall monetised cost of the peer review system, including application preparation, has been estimated to account for as much as 20–35 per cent of the allocated budget ( Gluckman, 2012). Graves *et al.* (2011) report that the monetised costs of the application system for NHMRC are $14,000 per grant, whilst extrapolating RCUK ( Research Councils UK, 2006) estimates suggests that the costs of the application process are 10–17 per cent of the total cost of research. An evaluation of the CIHR Operating Open Grants Program (OOGP) found the application cost of OOGP grants to be Can$14,000 ( Peckham *et al.*, 2012). A detailed review of preparing grants for NIH for nursing research reports similarly high costs to institutions ( Kulage *et al.*, 2015). When providing congressional testimony individual researchers have estimated that as much as 60 per cent of their time is devoted to seeking funding ( Fang & Casadevall, 2009).

***Burden on applicants.*** The bulk of the resources consumed by the peer review process are in the writing and reviewing of applications. RCUK work showed the distribution of monetised burden was 74 per cent in application production, 21 per cent in reviewing process (including time of reviewers, panel membership and modifying proposals), and 5 per cent in Research Council costs and payments to reviewers ( Research Councils UK, 2006).

More recent work by Graves *et al.* (2011) used a small survey of NHMRC researchers to estimate that the burden fell even more heavily on the applicants, assigning a split of 85 per cent for application production, 9 per cent for reviewing and 5 per cent for administration. Barnett *et al.* (2015) reinforced this conclusion with a larger survey of 285 applicants who had submitted 632 proposals to four health services research funding rounds from May 2012 to November 2013, at the Australian Centre for Health Services Innovation. A review by the New Zealand Royal Society made a similar estimate of the burden shouldered by the applicants – pegging it at 80 per cent ( Gluckman, 2012).

In contrast two studies of the Natural Sciences and Engineering Research Council (NSERC) of Canada peer review process and came to strikingly different conclusions. Gordon & Poulin (2009) estimated the cost of the NSERC system, including application preparation, review and administration costs at Can$44m. They suggest this money could alternatively provide all researchers in the field with an annual baseline grant of Can$30,000. However, Roorda (2009) takes issue with Gordon and Poulin’s assumptions suggesting they have overestimated costs by a factor of 23. The correct answer appears to be in between – there is disagreement about how the costs should be allocated and neither side provides a justification of their estimates of the time spent on grant preparation (the key driver).

Herbert *et al.* (2013) suggest burden on NHMRC applicants could be reduced by simplifying the application process (currently 80–120 page applications). Other examples of funding agencies reducing the length and complexity of applications include NIH did cut the length of their applications for R01s ^8^ from 25 pages to 12 in 2009, although there were calls to make the application even shorter ( Fang & Casadevall, 2009).

Barnett *et al.* (2015) examined the effect of reducing the complexity of the application. Surprisingly, they found that reducing application complexity slightly increased preparation time. They suggest that this may be because researchers allocate a fixed fraction of their time to application preparation. Theoretical work by Geard & Noble (2010) using agent based modelling found that applicants devote ‘excessive’ time to proposal preparation ( Geard & Noble, 2010). Barnett *et al.* (2015) examined four rounds of a funding scheme in Australian which significantly shortened the application (to 1,200 words). Qualitative feedback was positive, suggesting it took seven days to develop an application, but generalisability is limited. The level of effort devoted to application preparation is all the more striking given Herbert *et al.*’s (2013) finding that increased effort did not translate into increased success rates.

A few qualitative studies have examined the burden of the system on particular groups of researchers and the wider implications on researchers’ quality of life. A survey of 215 NHMRC applicants concluded that the ‘impact of preparing grant proposals for a single annual deadline is stressful, time consuming and conflicts with family responsibilities’ (p.1), although it did not quantify the effects or time taken ( Herbert *et al.*, 2014). A study of early career investigators applying for funding at CIHR identified the application process as burdensome and noted the decrease in success rates for open operating grants from 30 per cent in 2005–2006 to 15 per cent in 2014–2015 ( Association of Canadian Early Career Health Researchers, 2016).

The institutional costs of application preparation were examined by the US Government Accountability Office (GAO) in 2016, which concluded that pre-award requirements for applicants to develop and submit detailed documentation for grant proposals, and increased prescriptiveness of certain requirements, had increased universities’ workload and costs, but the study ( GAO, 2016) did not quantify these increases

***Burden on reviewers and panel members.*** Time invested by reviewers and panel members is consistently identified as the second-highest monetised cost of peer review, making up about 15 per cent of the burden. Two types of studies carried out in this area have both aimed at optimising the process, balancing the trade-off between burden and quality to achieve efficiency.

The first study approach trialled simplified processes for grant review to test how much time they save and whether they affected funding decisions ( Herbert *et al.*, 2015) – particular the use of a shortened application form and smaller review panels. They found the simplified processes achieved agreement with the current award system of close to 75 per cent (which they suggested was the ‘acceptable’ threshold based on a review of previous surveys), at estimated savings of 33–78 per cent of review costs.

The second study used statistical techniques to estimate the optimum number of reviewers ( Snell, 2015) trading off improved reproducibility with additional reviewer burden. They found that five reviewers were optimal; similar work by Graves *et al.* (2011) on a different funding scheme found 11 reviewers was the most effective number.

In addition to experimental changes there are examples of funding agency policy changes that have been examined. The NSF changed its review procedures in 2012 to reduce burden by introducing triage on short preliminary applications with a 75 per cent cull rate, with annual rather than six-monthly applications. The General Accountability Office has praised the system and it reduces administrative burden on programme officers. However, because several changes happened simultaneously, it is not clear whether this is because of the triaging. It also resulted in reduced success rates, partly because of more applications (perhaps because they were easier to write but also because of funding reductions ( Mervis, 2016).

One of the drivers of the burden on funders is identifying appropriate reviewers for each proposal. Mervis (2014) reports on a radical experiment at NSF where applicants reviewed each other’s grants (each applicant completing seven reviews), consequently reducing this burden to zero. To guard against applicants marking their competitors down, they were rewarded for scores that aligned with the other reviewers. The pilot allowed the number of reviews per proposal to be increased from three or four to seven and the reviews provided were more detailed. Because of the additional reviews, NSF was able to dispense with panel discussion, thus saving administrative costs.

## Discussion

In this section we summarise our findings: firstly, on the availability of evidence, considering the scope and coverage of the existing literature; secondly, on what that the evidence shows, and finally, highlighting the implications for health research funders.

### Availability of evidence

Questions about the effectiveness and burden of peer review can be addressed at two levels. At a high level, does peer review support valuable science? And at a lower level, can the design of peer review systems be improved to increase effectiveness and reduce burden?

It is clear that the current system of funding has produced significant benefits for society, suggesting that peer review supports valuable science. However, whether peer review is demonstrably better than any other system is impossible to judge with certainty because of the lack of comparators: no funding agencies have made significant use of alternative systems.

Moving to the lower level, considering comparisons between or research on peer review systems, there is only a very small number of robust, well-conducted studies. Much of the literature identified is anecdotal in nature and we found no systematic reviews, underlining the fragility of the evidence base. However, we did identify a series of robust, high-quality studies that have been carried out since our last review in 2009. Despite this new work it is still true that most studies examine the peer review process of one particular funder in one particular context, rather than looking across funders or contexts, and few go beyond process measures to judge effectiveness.

This persistent lack of evidence about the allocation of the ‘inputs’ to research is all the more striking given the advances in understanding the outputs and outcomes of research through research impact assessment over the last decade.

### Findings from the available evidence

The central problem when assessing peer review is the lack of an absolute standard or ‘ground truth’ to judge against. There will be uncertainty in all peer review decisions - it is, after all, predicting the future. And there is evidence suggesting it is not a particularly good predictor, at least for bibliometric performance. At present most funders do not capture, use, or even acknowledge this uncertainty, despite clear evidence of inconsistency in peer review ratings and mixed evidence on the reproducibility of panel decisions.

These is good evidence that peer review suffers from biases. The strongest evidence is of a bias against innovation and although a range of improvements have been suggested, none have been robustly evaluated. There is some evidence peer review is influenced by cognitive distance and suffers from cronyism and suggestive evidence that there are age biases. Considerable work has been done on gender bias, with conflicting results, which illustrates the challenges of accounting for biases outside the scope of the peer review process, for example through eligibility or the culture of the wider scientific system.

Though the problem of burden is widely recognised, funders’ considerations often focus on their own and reviewers’ burden as these are more immediately visible (and costly) to them. However, it is clear that the burden largely falls on applicants (rather than reviewers or panel members).

Falling success rates across many funders compound the burden on applicants. One way to address these challenges could be to reduce the complexity of the application process, with evidence suggesting similar decisions can be made with much shorter applications and less information. However, small decreases in application length do not seem to translate into application preparation time so such changes would need to be carefully evaluated.

Despite the plethora of comment pieces criticising the peer review system, there is no empirical evidence suggesting whether peer review has more or less support among key stakeholders than it did in 2009.

### Potential improvements

***Improving effectiveness.*** This section outlines our reflections on ideas for improving peer review processes. We concentrate on ideas that augment or refine peer review – as those approaches were most comprehensively covered by our search approach. Other approaches that are more complete alternatives to peer review for example peer to peer allocation were beyond the scope of this review ( Bollen *et al.*, 2017).

We feel the uncertainty in peer review - clear in the inconsistency of ratings and weak predictive power in terms of future academic performance - should be acknowledged, captured and used to improve decision making and for analysis. Reviewers should be asked both for their rating of the proposal and a measure of their confidence in this rating - some smaller funders, such as the Villum and Velux Foundations in Denmark, are starting to implement such systems. Funders could also analyse levels of disagreement between reviewers, which may be an indicator of innovative research ( Linton, 2016), or take a portfolio approach selecting projects scoring highly across different criteria, including innovation ( Lee, 2015).

A second approach is to acknowledge the difficulty of predicting the future and introduce an explicit element of randomness into the allocation system. This could be done to differing extents – from completely random allocation of funding to the use of a lottery system within set groups of applicants. Fang & Casadevall (2016) propose a two-stage system, in which the best applications are identified and then a smaller percentage are funded using a lottery. Avin (2015) proposes using two thresholds, above the higher threshold all applications are funded and below the lower threshold all applications are rejected, applications between the two thresholds are funded at random, effectively blurring the funding line.

A lottery approach should reduce biases in decision making since the selection from the fundable pool is random; however, applicant eligibility restrictions/selection for the lottery could reintroduce bias. Selecting into a fundable pool requires less fine-grained decisions addressing concerns about the reliability of peer review. The use of lottery systems is a promising, but politically challenging idea, so far is has only been used in very limited cases, such as the Explorer Grants offered by the Health Research Council of New Zealand; the Seed Projects offered by Science for Technological Innovation also in New Zealand and the Experiment! Grants from the Volkswagen Foundation ^9^, and as such we think using elements of lottery allocation merits further empirical research ( Barnett, 2016). Complex approaches combining assessment and lottery, although theoretically attractive, suffer from the disadvantage of sacrificing understandability ( Kurokawa *et al.*, 2015).

Other approaches to address bias include blinding of reviewers (e.g. Lee *et al.*, 2012), though the feasibility of this is debated ( Bhattacharjee, 2012). More practically funders have also used training approaches to address bias (e.g. CIHR) and to improve quality of reviews (e.g. NIH, 2008) and there is limited evidence that the approach could reduce the discrepancies between reviewers ( Sattler *et al.*, 2015).

***Reducing Burden.*** Applicant burden should be considered as a priority compared to reviewer and administrative burden as it represents around 75% of the system burden. This can be addressed by reducing the level of burden or increasing the value unsuccessful applicants receive by applying. Changes to reduce burden need to be carefully evaluated as there is evidence that even significant reductions in application length/complexity may not reduce applicant burden as much as expected. An alternative approach is to make the process more valuable for the applicants. Reviewer and panel feedback may be one way to do this (although one of the reviewers of this paper noted the concern that providing feedback may open a funder to appeals from rejected applicants).

Technology provides ways to reduce the time burden of the peer review process for panel members and funders - for example by eliminating travel - and does not appear to significantly affect the outcomes. However, face-to-face discussion of applications brings other side-benefits, including social interaction and network formation, other research suggests these side-benefits may be important to the progress of science and hence may need to be supported in other ways if peer review is done remotely.

Altering the format of research proposals to incorporate multi-media or video has been suggested as a way to improve information transmission and reduce burden, but the effects of doing so have not been tested ( Doran *et al.*, 2014).

***Improving the evidence base.*** It remains striking how little robust evidence is available about peer review as a method for grant allocation. Given the centrality of the peer review process in the current science funding system, there is a need for better evidence, not only on the overall effectiveness of peer review but also to help improve the design of peer review processes. We suggest three fruitful areas for investigator are the links between the peer review process and the wider context of science funding; the social processes of peer review and panel meetings.

System changes (such as the overall amount of funding) affect the peer review process, and peer review changes affect the system, so both need to be considered together to understand the dynamic behaviour of the overall research process. Nearly all of the studies we identified considered aspects of the peer review system in isolation – for example tracking success rates or reviewer burden. However, system changes such as decreased funding, or changes in researcher demographics, often happen alongside, and interact with, changes to the peer review system. To address these questions may require developing the modelling and simulation approaches such as those in Avin (2015), Geard & Nobel (2010) and Höylä *et al.* (2016).

Even in the fairly barren landscape of evidence we explored, it was startling that we could find no studies examining the social processes that occur during panel discussions – a central part of the peer review process. Such studies will clearly be challenging and require the cooperation of funders working in concert, but we feel are essential to understand how to optimise one of the fundamental processes of science.

At a more mundane level, funders should be more willing to experiment with, evaluate and publish results from evaluations of alternative approaches. Through our conversations with funders it appears that where analysis is carried out it is often not published, partly because of the extreme sensitivity around funding allocation procedures. Funders are not the only ones who need to take a more reflective approach: they will need the support of the wider scientific community to support such investigations, and acknowledge the lack of evidence about the primacy of the current system and the impossibility of achieving perfection.

## Conclusions

Many criticisms of the peer review system reflect conflicts between the needs of stakeholders. Researchers look to peer review to uphold research standards and promote the ‘best’ science, while politicians and funders use it to provide accountability for spending ( Viner *et al.*, 2004). This tension requires peer review to both protect the identities of reviewers while appearing transparent to applicants; to be innovative yet assure quality; to be based on human judgement yet free of human biases ( Hackett & Chubin, 2003).

We think that current dissatisfaction with the peer review process is amplified by falling success rates, so it is important to remember that the concerns around peer review are heavily influenced by funding policy and the size of research budgets.

As a society, if we are to improve how we use our research funds, we need a better understanding of the peer review process. When making changes, funders should: build in before and after comparisons; strive to make data available for analysis; openly publish studies of their processes and work together on comparative analysis.

We need to overcome the reluctance of funders and scientists to acknowledge the uncertainties intrinsic to allocating research funding, and encourage them to experiment with peer review and other allocation processes.

## Data availability

All data underlying the results are available as part of the article and no additional source data are required.

## Notes

^1^GRADE is an internationally accepted system for the assessment of evidence quality. GRADE offers four levels of evidence quality: high, moderate, low, and very low. Randomised trials begin as high-quality evidence and observational studies as low-quality evidence, and studies may be downgraded as a result of limitations in study design or implementation, imprecision of estimates, variability in results, indirectness of evidence, or publication bias. Equally, quality may be upgraded based on a very large magnitude of effect or if all plausible biases would reduce an apparent effect ( Guyatt *et al.*, 2008).

^4^As of 5 January 2017: https://www.macfound.org/programs/fellows/

^5^Defined as ‘the correlation between two independent assessors of the same submissions across a large number of different submissions’ ( Jayasinghe *et al.*, 2003, p.280).

^6^In a multi-stage review process, the assessor at each evaluation stage will know the score given to a particular research proposal at the previous stage. This particular study assessed the reliability of grant peer review processes by determining the proportion of those applications for which the dependent ratings on the same proposal did not change from the first to the second and third stage.

^7^ Bornmann *et al.* (2007) are clear, however, that the reasons for this observed discrepancy are not known. This is important because aggregation effects over a range of fields of study *may* – as the authors acknowledge –create strong statistical effects implying gender bias. The authors also suggest that future improvements to the model will need to take into account the cohort of application, since the study described here covered publications produced over the period 1979–2004, and there have been significant changes to reduce gender bias in science and science funding over this period.

^8^The Research Project Grant (R01) is the original and historically oldest grant mechanism used by NIH. The R01 provides support for health-related research and development based on the mission of the NIH. R01s can be investigator-initiated or can solicited via a Request for Applications.

^9^Web sites accessed on 13 February 2018: Explorer grants: http://www.hrc.govt.nz/funding-opportunities/researcher-initiated-proposals/explorer-grants; Seed projects: http://www.sftichallenge.govt.nz/research/seed-projects; Experiment! Grants: https://www.volkswagenstiftung.de/en/funding/our-funding-portfolio-at-a-glance/experiment.html
