# Supplementary material for: What do we know about grant peer review in the health sciences?
Source: F1000Res. 2018 Mar 27;6:1335. Originally published 2017 Aug 7. [Version 2] doi: 10.12688/f1000research.11917.2 (PMC5883382; doi:10.12688/f1000research.11917.2)
Supplement: Peer review history

# Peer review: Referee response for version 2

**DOI:** 10.5256/f1000research.15277.r32530

**Published:** 2018-03-29

**Authors:** Adrian Barnett

**Affiliations:** 1 Institute of Health and Biomedical Innovation, Queensland University of Technology, Kelvin Grove, Qld, Australia

The authors have answered my previous queries. This will be a useful paper for those looking for evidence for funding peer review.

 I wouldn't say that the NSF approach "reduced the burden to zero" (page 11, second column) as the researchers still needed to spend time on their reviews. It was an interesting approach and could be more efficient. Plus it avoids researchers only "taking" from the system and not giving back.

 Minor comments

- Page 7, first column, space missing in "decisionmaking"
- Page 9, first column, “decreases in early career” add “in”
- Page 9, citations to Wenneras (first column) and Bornmann (second column) should have brackets around the year not the authors
- Page 10, second column, “include NIH did cut” needs re-wording
- Page 10, second column, last paragraph, “Australian” should be “Australia”
- Page 11, last full paragraph in first column, open bracket with no closing bracket
- Page 11, second column, last paragraph, Donald Braben suggested it be called “peer preview” rather than “peer review” because reviewers try to predict the future ^1^
- Page 13, second column, our group has recently published a qualitative study of funding peer review panels that included observations on the dynamics between panelists ^2^

I have read this submission. I believe that I have an appropriate level of expertise to confirm that it is of an acceptable scientific standard.

## References

[1] BrabenD: Promoting the Planck Club: How Defiant Youth, Irreverent Researchers and Liberated Universities Can Foster Prosperity Indefinitely. *Wiley-Blackwell*.2014;

[2] CoveneyJHerbertDLHillKMowKEGravesNBarnettA: 'Are you siding with a personality or the grant proposal?': observations on how peer review panels function. *Res Integr Peer Rev*.2017;2: 10.1186/s41073-017-0043-x 19 10.1186/s41073-017-0043-x 29451548PMC5803633

# Peer review: Referee response for version 1

**DOI:** 10.5256/f1000research.12882.r24828

**Published:** 2017-08-14

**Authors:** Aled Edwards

**Affiliations:** 1 Structural Genomics Consortium, University of Toronto, Toronto, ON, Canada

This is a nice and comprehensive review of the studies that assess the strengths and weaknesses of the peer review process.

 Although there are no new conclusions, it does reveal or highlight the three major issues that plague our mechanism to dole out research funding and the use of peer review. 

 The first is fundamental - without a clear and agreed upon definition of what constitutes the "best" science, or the "best" outcomes, it will be impossible to know whether the grant adjudication process is meeting, or can ever meet, its objectives. 

 Second, the paper highlights what is often overlooked, and this is the real cost in time of writing funding proposals.  Although scientists often manufacture a narrative that writing grants is good for them (for example, making you catch up on the literature), the real costs to the system are rarely factored in.   I think this paper did a nice job in highlighted this issue.

 Finally, I would have liked a greater discussion on what is an amazing disconnect.   All the empirical evidence highlights the deficiencies of the process to allocate grant funding.  It is clear that it is neither scientifically founded, nor evidence-based.   Yet one of the only strongly supported aspects of the peer review process is that is has the strong support of the community.  I find this fascinating.

I have read this submission. I believe that I have an appropriate level of expertise to confirm that it is of an acceptable scientific standard.

# Response

**Published:** 2018-03-19

**Authors:** Steven Wooding

**Affiliations:** University of Cambridge, UK

Thank you for your review and comments. Reflecting on the disconnect you highlight in the final paragraph above we have added a sentence to the paper commenting on this and noting it as a topic that might warrant further investigation.

# Peer review: Referee response for version 1

**DOI:** 10.5256/f1000research.12882.r24827

**Published:** 2017-08-09

**Authors:** Adrian Barnett

**Affiliations:** 1 Institute of Health and Biomedical Innovation, Queensland University of Technology, Kelvin Grove, Qld, Australia

This is a very useful review of an area that is vitally important for science and that is constantly being examined by funding agencies. It included some papers that I had not read, but there were a few additional papers that I thought could be included (detailed below).

 The results once again highlight the incredible lack of studies in this area. The paper ends with some sensible recommendations, including the need for funders to experiment more and make their data available to researchers.

 I was surprised that some of the more innovative solutions to funding peer review were not included, specifically using prediction markets ^1^ and using the “wisdom of the crowd” ^2^.

 Why was 2009 chosen as the time threshold? Is it because that was the year of the previous review?

 This paper should be included in the discussion on interdisciplinary research: Interdisciplinary research has consistently lower funding success, Lindell Bromham, Russell Dinnage & Xia Hua.  Nature  534, 684–687 doi:10.1038/nature18315 ^3^. This paper agrees with the other two mentioned, as there are lower success rates for applications with more cross-disciplinary researchers.  

 One force against “cognitive particularism” is that strict conflict of interest rules from funding bodies can often rule out reviewers with the greatest knowledge, particularly in small fields or small countries. This study may touch on this issue: Abdoul H *et al*, Non-Financial Conflicts of Interest in Academic Grant Evaluation: A Qualitative Study of Multiple Stakeholders in France, PLoS ONE,  7/4: e35247. ^4^

 In terms of using technology in the review process, some researchers have suggested that videos may produce more reliable peer reviewer ratings and take less time to prepare: Doran MR, Lott WB, Doran SE. Trends Biochem Sci. 2014 Apr;39(4):151-3. doi: 10.1016/j.tibs.2014.01.004. Multimedia: a necessary step in the evolution of research funding applications ^5^.

**Minor comments**

- Introduction, 1st paragraph. As an Australian researcher I would argue that the ERA has not really measured research quality, rather it has simply measured research output. Maybe you could say “Funders have attempted to gather evidence…”
- The “>95%” figure in the abstract feels about right, but is there a reference for this?
- For the Google search terms, “fellowship” could also have been added, so “Fellowship OR funding”.
- The link to this paper did not work: The Novelty Paradox & Bias for Normal Science: Evidence from Randomized Medical Grant Proposal Evaluations
- Page 6, “when reviewing research closer to application”, I didn’t understand this.
- Page 7, “only affects the funding decision for around 10 per cent of applications relative to original scores” but that could still be an important percentage, particularly if it’s those near the funding line
- Page 9, “found that panel assessments of full proposals and shorter anonymised versions of the same proposals showed weak correlations” do you need to add, “implying that knowledge of the applicants influences the score”. Although as well as a change in blinding there was also a change in the size of the application, so it may be hard to conclude anything about cronyism here.
- Footnote 9, the NZ Health Research Council has been using random allocation for this scheme since at least April 2015
- I agree that giving more feedback would improve the value of the process (page 12), but our experience with the NHMRC is that this also opens them up to appeals which can take a lot of time for their staff. This doesn’t mean that we shouldn’t try, and giving frank feedback was a feature of a funding scheme we designed (already cited paper “Streamlined research funding using short proposals and accelerated peer review: an observational study”).
- This paper may be of interest: Scientometrics, July 2016, Volume 108, Issue 1, pp 263–288, The consequences of competition: simulating the effects of research grant allocation strategies. ^6^
- This parody of a grant application may be useful for the section on peer reviewers being biased against innovative proposals: doi:10.1097/ede.0000000000000453 “John Snow’s Grant Application” ^7^
- This paper examined the costs of applying for NIH funding and could be included in the section on the costs to applicants: Nursing Outlook, Volume 63, Issue 6, November–December 2015, Pages 639-649, Time and costs of preparing and submitting an NIH grant application at a school of nursing, https://doi.org/10.1016/j.outlook.2015.09.003 ^8^

I have read this submission. I believe that I have an appropriate level of expertise to confirm that it is of an acceptable scientific standard, however I have significant reservations, as outlined above.

## References

[1] MunafoMRPfeifferTAltmejdAHeikenstenEAlmenbergJBirdAChenYWilsonBJohannessonMDreberA: Using prediction markets to forecast research evaluations. *R Soc Open Sci*.2015;2(10) : 10.1098/rsos.150287 150287 10.1098/rsos.150287 26587243PMC4632515

[2] BollenJCrandallDJunkDDingYBörnerK: An efficient system to fund science: from proposal review to peer-to-peer distributions. *Scientometrics*.2017;110(1) : 10.1007/s11192-016-2110-3 521-528 10.1007/s11192-016-2110-3 PMC596371929795961

[3] BromhamLDinnageRHuaX: Interdisciplinary research has consistently lower funding success. *Nature*.2016;534(7609) : 10.1038/nature18315 684-687 10.1038/nature18315 27357795

[4] AbdoulHPerreyCTubachFAmielPDurand-ZaleskiIAlbertiC: Non-financial conflicts of interest in academic grant evaluation: a qualitative study of multiple stakeholders in France. *PLoS One*.2012;7(4) : 10.1371/journal.pone.0035247 e35247 10.1371/journal.pone.0035247 22496913PMC3322153

[5] DoranMRLottWBDoranSE: Multimedia: a necessary step in the evolution of research funding applications. *Trends Biochem Sci*.2014;39(4) : 10.1016/j.tibs.2014.01.004 151-3 10.1016/j.tibs.2014.01.004 24703407

[6] HöyläTBartneckCTiihonenT: The consequences of competition: simulating the effects of research grant allocation strategies. *Scientometrics*.2016;108(1) : 10.1007/s11192-016-1940-3 263-288 10.1007/s11192-016-1940-3

[7] RothmanK: John Snow’s Grant Application. *Epidemiology*.2016;27(3) : 10.1097/EDE.0000000000000453 311-313 10.1097/EDE.0000000000000453 26829161

[8] KulageKMSchnallRHickeyKTTraversJZezulinskiKTorresFBurgessJLarsonEL: Time and costs of preparing and submitting an NIH grant application at a school of nursing. *Nurs Outlook*.63(6) : 10.1016/j.outlook.2015.09.003 639-49 10.1016/j.outlook.2015.09.003 26566959

# Response

**Published:** 2018-03-19

**Authors:** Steven Wooding

**Affiliations:** University of Cambridge, UK

Thank you for your review and helpful comments. We have made some revisions to the paper in response to these suggestions. In particular, we have added most of the references you suggest to the paper and made some clarifications where required. We have also updated methods with the reason for the 2009 cut off and run searches using the term ‘fellowship’ as suggested, which added two additional papers to the review. Responses to main specific points raised are as follows:

 ·         Prediction markets and “wisdom of the crowds”: These approaches are complete alternatives to peer review, rather than refinements, and hence are beyond the scope of the review. The wisdom of the crowds approach is for ex post evaluation rather than ex ante – it simulated REF2014 assessments.

 ·         2009 time threshold: Yes, this is because it was the date of the previous review.

 ·         Paper by Bromham et al: Added

 ·         Conflict of interest rules: Interesting point, but we don’t have any evidence which would enable us to comment on this in detail. The study by Abdoul et al  you note mainly concerns the awareness that reviewers have of conflicts of interests rather than the effects of these conflicts in terms of excluding knowledgeable reviewers

 ·         Use of videos: Added a note that this approach has been suggested but not yet evaluated.

 ·         Minor comments also all addressed as appropriate within the revised paper.
